# Acne caused by ziprasidone in a young patient with bipolar disorder: A case report

**DOI:** 10.3389/fpsyt.2022.948977

**Published:** 2022-11-04

**Authors:** Yiwen Yuan, Xiaoqing Li, Xingmei Jiang, Zhixiong Li, Ying Ou, Zhe Li

**Affiliations:** ^1^Mental Health Center, West China Hospital, Sichuan University, Chengdu, China; ^2^People's Hospital of Jianyang City, Chengdu, China; ^3^West China School of Nursing, Sichuan University, Chengdu, China; ^4^Department of Gastroenterology, West China Hospital, Sichuan University, Chengdu, China; ^5^Sichuan Clinical Medical Research Center for Mental Disorders, Chengdu, China; ^6^The Third Department of Clinical Psychology, Karamay Municipal People's Hospital, Karamay, China

**Keywords:** ziprasidone, acne, bipolar disorder, adverse reaction, case report

## Abstract

**Background:**

Ziprasidone is a second-generation antipsychotic drug commonly used to treat schizophrenia and bipolar disorder. Acne is a common inflammatory disease of sebaceous glands in adolescents that is often co-morbid with anxiety and depression, which may reduce treatment compliance. Through unknown mechanisms, ziprasidone may cause a range of inflammatory responses. Whether ziprasidone can cause acne in young patients with bipolar disorder has not been reported.

**Case summary:**

We report a 23-year-old woman with a 5-year history of bipolar disorder who experienced acne during use of ziprasidone. She was admitted to our hospital during 1-month aggravation of her symptoms and was diagnosed with bipolar I disorder (current or most recent episode of depression) with psychotic features. She was given ziprasidone and soon developed acne, which she never had before; the rash worsened substantially when the ziprasidone dose was increased. At the same time, levels of inflammatory factors increased. The rash resolved after ziprasidone therapy was stopped.

**Conclusion:**

When prescribing ziprasidone to young people with bipolar disorder, clinicians should consider the potential for adverse skin reactions. It may be useful to assay levels of inflammatory markers during ziprasidone therapy and adjust the dose if necessary in order to ensure treatment compliance.

## Introduction

Ziprasidone is a second-generation antipsychotic drug commonly used to treat schizophrenia and manic episodes of bipolar disorder (1, 2). Ziprasidone exerts antidepressant effects by acting as a 5-HT/D antagonist, 5HT1D receptor agonist, and inhibitor of norepinephrine/serotonin (3, 4). A systematic review and exploratory meta-analysis concluded that ziprasidone and other second-generation antipsychotics can alleviate depressive symptoms in patients with bipolar disorder (5). Ziprasidone is generally well tolerated: it is unlikely to alter glucolipid metabolism because it weakly antagonizes the activity of M1 and H1 receptors, it does not substantially affect body weight, and it does not excessively sedate the individual (6–8). Despite its safety and efficacy, ziprasidone often prolongs QTc, which has been associated with higher risk of Torsades de Pointes tachycardia and cerebrovascular events (4, 9). The drug is also associated with extrapyramidal symptoms, headache, and insomnia (10), as well as inflammatory responses that may be related to allergic reactions (11).

Acne is a common inflammatory disease of sebaceous glands in adolescents. It often produces undesired skin lesions and can even disfigure, reducing quality of life. Patients with acne often simultaneously suffer anxiety and depression (12).

We are unaware of reports that ziprasidone can cause acne in young patients with bipolar disorder. Here we describe a young woman with bipolar disorder who developed acne on ziprasidone therapy.

## Case presentation

### Chief complaints

The patient, a 23-year-old woman, was admitted to our hospital in November 2021 because of alternating episodes of depression and excitement, a condition that had persisted longer than 5 years and had worsened in the preceding month.

More than 5 years before the current admission, our patient began to manifest progressive depression of mood and energy level, loss of interest and ability to concentrate, memory loss, lazy talking, pessimism and negativity, including suicidal thoughts. Within 6 months, loss of appetite led her to lose 5 kg of body weight. At that time, she was hospitalized at our center for 12 days, diagnosed with major depressive disorder (single episode, severe) based on the 5th edition of the Diagnostic and Statistical Manual of Mental Disorders (DSM-5) (13) and treated with unrecorded doses of sertraline. She was discharged when her condition seemed to be stable with regular medication.

At 4 years before her current admission, she unexpectedly experienced elevated emotions and inflated self-esteem, more positive self-opinion, and decreased need for sleep, with no apparent trigger or explanation. She was also more talkative than usual and sometimes could not stop talking. She would often lose focus in class. These symptoms lasted for about half a year, when she again showed depressed mood, lack of interest, loss of energy, and irritability. She also felt diminished interest in all activities most of the day. Nearly every night, she had difficulty falling asleep. She felt feelings of worthlessness and had recurring thoughts of death. She sometimes stabbed her thighs with a pen when she was upset, and she sometimes hit her head against the wall to vent emotions. She was hospitalized again at our center and was diagnosed with bipolar I disorder (current or most recent episode of depression, severe) based on the DSM-5 (13). She was given sertraline (100 mg, once a day) and quetiapine (0.15 g, once a night), and she was discharged after 14 days of treatment. After discharge, the patient took her medicine intermittently and her condition was unstable: her mood alternated between good and bad over the course of a few days.

At 11 months before the current admission, her outpatient medication was adjusted to sertraline (100 mg, once a day) and quetiapine (0.2 g, once a night), but she still took it intermittently. She became depressed and agitated again, and she felt low energy and would wake up too early. She indicated that she did not want to live anymore, wrote a suicide note, took several sleeping pills of unknown formulation and dose, and cut her wrists. Her parents sent her to a local hospital, where her wounds were sutured and she was also diagnosed with bipolar I disorder (current or most recent episode of depression, severe) based on the DSM-5 (13). She was given sertraline (150 mg, once daily), quetiapine (0.3 g, once per night), and lithium carbonate (0.25 g, twice per day). In addition, she received modified electroconvulsive therapy eight times. She was discharged 3 weeks later, when she showed no significant abnormality by electrocardiography or routine assays of blood, liver and kidney function, electrolytes, inflammatory indicators, or stool. The blood level of lithium carbonate was 5.68 μg/ml (reference value: 4.16–8.32 μg/ml).

At 3 months before the current admission, the patient gained about 4 kg and stopped taking her medication. She became excited, energetic, and sensitive; she began to spend money extravagantly; and she began to believe that others were talking about her. Her medication was adjusted to ziprasidone (40 mg, once a night) and lithium carbonate (250 mg, three times a day). The patient was offered ziprasidone after she indicated that she wanted a medication unlikely to cause weight gain. After 1 week of therapy, scattered papules and pustules appeared on her face, with some exudation. The blood level of lithium carbonate was 4.57 μg/ml (reference value: 4.16–8.32 μg/ml). The QTc interval on electrocardiography was 435 ms (reference value: < 470 ms for women). She went to the outpatient dermatology clinic of our hospital, where her levels of inflammatory factors were found to be as follows: tumor necrosis factor-α (TNF-α), 4.3 pg/ml (reference, < 8.1 pg/ml); interleukin-1β (IL-1β), 14.1 pg/ml (0–5 pg/ml); interleukin-6 (IL-6), 2.70 pg/ml (0.00–7.00 pg/ml); and C-reactive protein (CRP), 5.02 mg/L (< 5 mg/L). She was diagnosed with acne and given vitamin A acid ointment to apply externally and minocycline hydrochloride capsules (50 mg) to take orally twice a day. The rash resolved after 2 weeks of treatment, when the levels of inflammatory factors were as follows: TNF-α, 8.2 pg/ml (reference, < 8.1 pg/ml); IL-1β, 12.0 pg/ml (0–5 pg/ml); IL-6, 2.80 pg/ml (0.00–7.00 pg/ml); and CRP, 4.80 mg/L (< 5 mg/L).

However, the patient remained excited and energetic, easily irritated, sensitive and suspicious. During follow-up in our outpatient department, the dosage of ziprasidone was adjusted to 40 mg twice a day. At this time, her levels of inflammatory factors were as follows: TNF-α, 5.0 pg/ml (reference, < 8.1 pg/ml); IL-1β, 10 pg/ml (0–5 pg/ml); IL-6, 3.00 pg/ml (0.00–7.00 pg/ml); and CRP, 6.00 mg/L (< 5 mg/L). After 1 week, the patient's facial acne had worsened, with papules and pustules, obvious exudate and pus. The blood level of lithium carbonate was 6.79 μg/ml (reference value: 4.16–8.32 μg/ml). The QTc interval was 447 ms (reference value: < 470 ms for women). The dermatology department continued to recommend vitamin A acid ointment and minocycline hydrochloride capsules (50 mg, twice a day).

The patient and her family members felt that the acne was related to her ziprasidone, so she stopped using it. After 2 weeks, the acne improved, and no exudation or pus appeared. However, her mood remained unstable.

At 1 month before her current admission, her condition worsened: she began to show depressed mood most of the day, minimal speech and laziness, pessimism and negativity. She also experienced loss of appetite and insomnia. She could not attend school and had suicidal thoughts; she attempted suicide several times by jumping off a building.

### History of past illness

Her medical history included an untreated, currently asymptomatic thyroid nodule that had originally been detected 11 months previously. She had been diagnosed with postural hypotension in January 2021, but she was currently asymptomatic. We found nothing else remarkable in her medical or family history. She denied a history of mental illness in the previous two or three generations, and she denied consuming alcohol or other psychoactive substances.

### Physical examination

Physical examination on admission revealed an old suture scar about 3 cm long on the left forearm, scattered acne on the face, and no other obvious abnormality. Neurological examination showed no abnormalities.

### Mental examination

Mental examination showed automatic admission, clean and appropriate dress, worried expression, self-care, passive contact, lack of concentration, self-awareness, and normal levels of intelligence and memory. She manifested several characteristics of depressive syndrome: low mood, decreased interest, energy, and activity; insomnia; as well as feelings of helplessness, worthlessness, indecisiveness, and excessive concern for physical health. She indicated a history of symptoms characteristic of manic syndrome: elevated emotions as well as high energy, inflated self-esteem, decreased need for sleep, distractibility, elevated activity, and greater talkativeness than usual. She did not report symptoms of anxiety syndrome, but she did report the following symptoms of hallucination and delusion: sensitivity, suspicion, and the feeling that others were talking about her. She also reported symptoms of sleep disorder, including difficulty in falling asleep, early waking, repeated waking during the night, and excessive dreaming. Impaired social functioning was evident.

### Laboratory examinations

The following routine examinations on admission revealed no obvious abnormalities: blood composition, liver and kidney function, electrolytes, tumor markers, pre-transfusion indicators, coagulation function, urine and stool. Levels of inflammatory factors were as follows: TNF-α, 4.5 pg/ml (< 8.1 pg/ml); IL-1β, 3.2 pg/ml (0–5 pg/ml); IL-6, 4.00 pg/ml (0.00–7.00 pg/ml); and CRP, 4.2 mg/L (< 5 mg/L). Thyroid ultrasonography showed uneven thyroid patterns potentially suggestive of Hashimoto's thyroiditis, as well as bilateral lobular thyroid nodules suggestive of nodular goiter. Computed tomography of the chest revealed small nodules in the lower lobe of the left lung, most likely of inflammatory origin. No obvious abnormalities were found by magnetic resonance imaging of the head, color ultrasonography of the heart or electroencephalography.

### Further diagnostic work-up

The patient scored 16 on the Hypomania Checking List-32, indicating a previous hypomania episode; 36 on the 24-item Hamilton Depression Scale (HAMD), indicating major depression; and 15 on the 14-item Hamilton Anxiety Scale (HAMA), indicating anxiety. Her total score on the Brief Psychiatric Rating Scale was 36, and her subscores were 8 for anxiety and depression, 12 for inactivity, 5 for thinking disorder, 3 for activation, and 8 for hostility and suspicion; these results suggested behavioral delay and impaired social functioning. She scored 6 on the Naranjo Causality Scale for determining the likelihood of an adverse drug reaction, indicating probable adverse drug reaction (14).

### Final diagnosis

Based on the patient's reported symptoms and test results, the patient was diagnosed with bipolar I disorder (current or most recent episode of depression with psychotic features) according to the criteria in the DSM-5 (13).

### Treatment

The patient was treated with quetiapine (0.3 g, once a night), lithium (500 mg twice a day), and agomelatine (50 mg, once a night). After consultation with the dermatology department, her acne was treated with minocycline hydrochloride capsules (50 mg, twice a day) and application of Bactroban (twice a day). During treatment, the patient's mood and her acne condition stabilized. By 2 weeks of treatment, the patient's mood was stable and her acne had improved substantially.

### Outcome and follow-up

**Figure 1:**
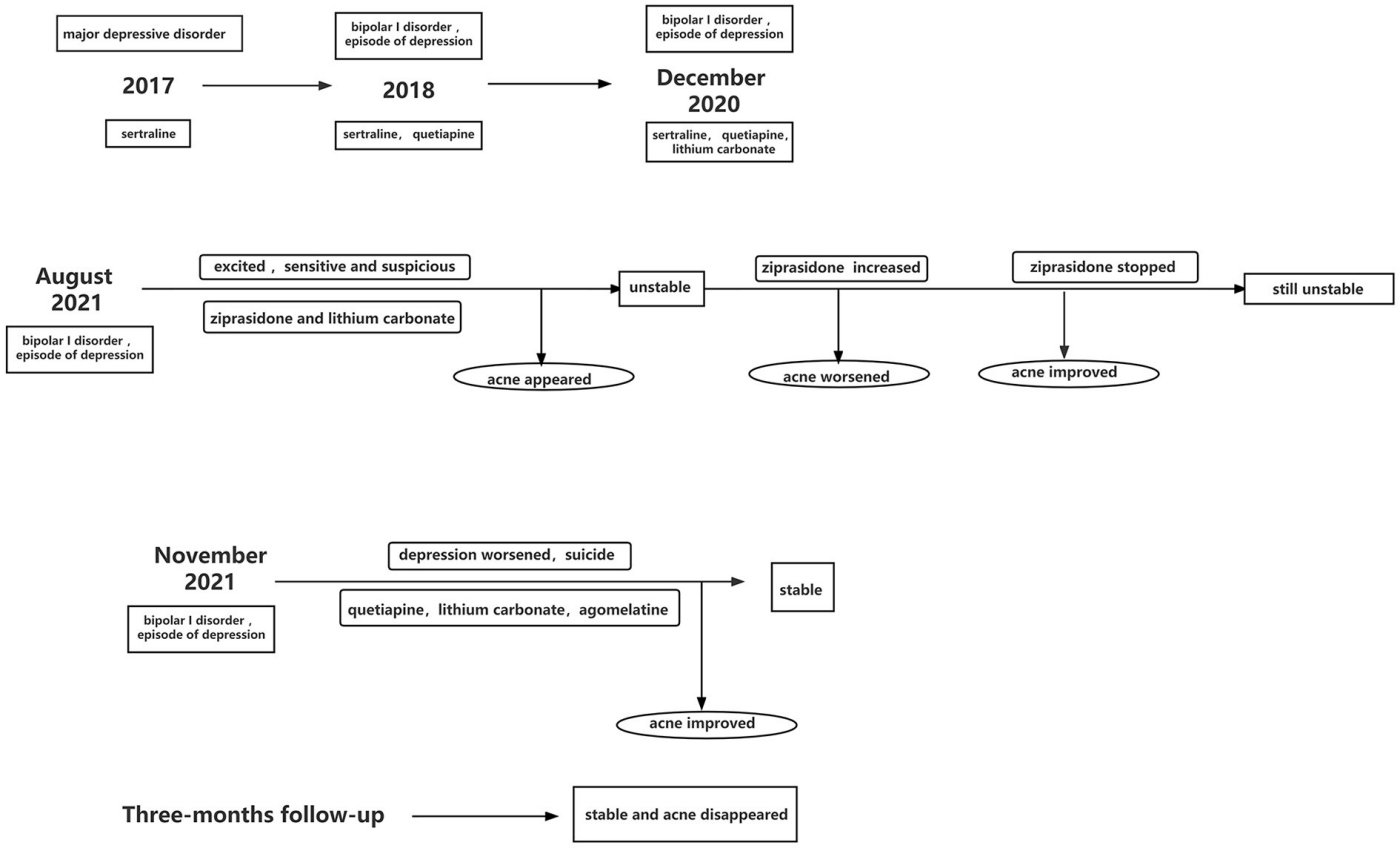
Historical and current information from this episode of care.

Follow-up at 3 months after discharge showed normal levels of TNF-α, IL-1β, IL-6, and CRP. She scored 6 on the HAMD, 5 on the HAMA and 5 on the Bech-Rafaelsen Mania Rating Scale. The patient's condition was stable, and acne did not reappear. She can go to school and had good tolerability of the medication. Historical and current information from this episode of care are shown in Figure 1.

## Discussion

Acne is a common inflammatory disease of the sebaceous glands, which may be caused by hormones such as insulin, insulin-like growth factor-1, and androgens; bacteria; adipogenesis; or pro-inflammatory lipids (15), which activate Toll-like receptor 2 on the surface of monocytes, triggering the release of pro-inflammatory cytokines (16). Acne manifests as pleomorphic skin lesions such as papules, pustules and nodules, and it usually occurs in teenagers. In fact, acne affects nearly all individuals between 15 and 17 years old to some degree, with negative impacts on their psychological and social life (17). The prevalence of acne tends to decrease with increasing age (18), although it can still occur in young adults. Bipolar disorder may lead to abnormal lipid metabolism through mechanisms involving inflammatory responses or altered insulin sensitivity, leading to acne (19). The age of our 23-year-old patient did not place her at high risk of acne, but her bipolar disorder may have.

Many drugs can cause acne, and such drug-induced acne often shows sudden onset and involves a rash of simplex inflammatory papules or papular pustules; patients can vary widely in age at onset. Drug-induced acne is typically diagnosed based on review of medical history while taking into account drug efficacy, treatment schedule and duration, as well as improvement after drug withdrawal in the absence of additional triggers (20).

Lithium carbonate is a mood stabilizer often used to treat bipolar disorder. Long-term use of lithium or high levels of it in the blood may lead to various skin diseases, such as psoriasis and acne, predominantly in men (21). Lithium-associated acne usually occurs after 2–6 months of lithium therapy (20, 22). Although our patient had a history of lithium carbonate therapy lasting more than 6 months, it did not trigger acne during hospitalization or follow-up. In addition, the level of lithium carbonate in her blood was in the normal range, and as a female, she was inherently at lower risk of lithium-associated acne. Therefore, we do not believe her acne was related to her lithium carbonate treatment.

Ziprasidone binds strongly to D2, D3, and α1-adrenergic receptors, and with lower affinity to 5HT2A, 5HT2C, and 5HT1D receptors. It also behaves as an agonist at the 5HT1A receptor (3, 4). Therefore it is commonly used to treat schizophrenia, bipolar disorder and depression. It shows only moderate affinity for the H1 receptor and does not show a clinically relevant interaction with the M1 receptor, implying minimal effects on metabolism or body weight (4, 23). On the other hand, it prolongs QTc, which may increase the risk of death and cerebrovascular events (4, 9) and can cause inflammatory allergic reactions (11), perhaps by promoting cell proliferation and the spread of macrophages through the blood; increasing levels of oxidizing molecules such as nitric oxide, superoxide, and reactive oxygen species; increasing the levels of proinflammatory cytokines TNF-α, interferon, IL-1, and IL-6; and regulating the expression of cytokine genes.

Our patient had a long disease course and repeatedly went to the doctor to adjust her medication, but she had no history of acne. Acne began to appear only after she began to take ziprasidone, and it worsened after the dose increased, which coincided with an increase in levels of inflammatory factors. The acne improved after ziprasidone therapy was stopped, and at the same time, the levels of inflammatory factors returned to normal. In addition, our patient developed a rash after combined administration of ziprasidone and lithium carbonate, and the rash worsened substantially after an increase in the ziprasidone dose, while it resolved after termination of ziprasidone. Given that our patient did not suffer rash when she took lithium carbonate alone, and given the timing between ziprasidone use and acne onset, we believe that the acne in our patient was the result of inflammatory responses caused by ziprasidone. Future studies should examine whether combining ziprasidone with lithium carbonate increases the risk of acne.

One case report has linked ziprasidone to subacute cutaneous lupus erythematosus (24). The US Food and Drug Administration has warned that ziprasidone may be associated with a rare, potentially fatal skin reaction called “drug reaction with eosinophilia and systemic symptoms” (DRESS). Symptoms of DRESS include fever, lymph node enlargement and inflammation of other organs such as the liver, kidney, lung, heart or pancreas (25). In contrast, our patient had normal eosinophil findings, and she showed no fever, lymph node enlargement, or other manifestations of inflammation in liver, kidney, lung, heart or pancreas. Our case highlights the need to clarify how ziprasidone may contribute to adverse skin reactions in patients with bipolar disorder.

Bipolar disorder has been linked to several physical comorbidities, such as cardiovascular disease, hyperlipidemia, metabolic disease, chronic pain, asthma, diarrhea, and acne (19, 26). Acne can easily be overlooked as an indicator of bipolar disorder, since the most frequent somatic comorbidities are cardiovascular disease, metabolic syndrome, migraine and infectious disease (27). Acne has also been reported as a co-morbidity in individuals with anxiety disorder or depression disorder (28). Psychiatric disorders may cause dermatosis because psychological stress hyperstimulates the amygdala, which activates the hypothalamic-pituitary-adrenal axis, stimulating release of pro-adrenocorticotropic hormone-releasing hormone, which in turn triggers degranulation in mast cells and permeabilizes the vasculature, ultimately leading to skin inflammation (29). Our case highlights the need to pay attention to the possibility of acne or other skin diseases in patients with bipolar disorder, in addition to the more frequent co-morbidities of cardiovascular, metabolic and other somatic diseases.

Optimizing treatment should take into account the patient's perspective (30, 31), especially since psychological distress in the patient can lead to dissatisfaction with treatment and lack of compliance (32). Our patient was satisfied while using ziprasidone, which she was recommended after indicating her desire for a medication unlikely to cause weight gain. However, she felt distressed when scattered papules and pustules appeared on her face while on the drug, and when these symptoms worsened after the dose was increased. During this treatment, we provided information about the nature and causes of bipolar disorder, medication, side effects and how to cope with daily problems. She remained satisfied with the treatment and showed good compliance.

## Conclusion

Acne should be considered as a potential adverse reaction when administering ziprasidone for bipolar disorder, especially to young patients. Inflammatory indicators should be checked during treatment, and the regimen should be adjusted if acne occurs in order to ensure treatment compliance and avoid harm to the patient's psychological state or more serious outcomes.

## Data availability statement

All the data on which the conclusions of this study are based are included in this article. Additional data is available from the corresponding author on reasonable request.

## Ethics statement

Written informed consent was obtained from the patient and her parents for the publication of any potentially identifiable data in this article.

## Author contributions

YY and XL wrote this case report. XJ, ZhiL, and YO provided clinical advice on patient management and contributed important intellectual content to the case report. ZheL was the patient's lead clinician and edited the case report for intellectual content. All authors approved the published version of the case report.

## Funding

This study was supported by grants to ZheL from the Applied Psychology Research Center of Sichuan Province (CSXL-202A08), Department of Human Resources and Social Security of Sichuan Province [(2020) 291-20], Science and Technology Bureau of Chengdu (2021-YF05-01336-SN), and Science and Technology Department of Sichuan Province (2022YFS0349). These funding agencies had no role in the design of the study; collection, analysis, or interpretation of the data; or writing of the manuscript.

## Conflict of interest

The authors declare that the research was conducted in the absence of any commercial or financial relationships that could be construed as a potential conflict of interest.

## Publisher's note

All claims expressed in this article are solely those of the authors and do not necessarily represent those of their affiliated organizations, or those of the publisher, the editors and the reviewers. Any product that may be evaluated in this article, or claim that may be made by its manufacturer, is not guaranteed or endorsed by the publisher.

## Abbreviations

DSM-5: Diagnostic and Statistical Manual of Mental Disorders, 5th Edition
DRESS: Drug Reaction With Eosinophilia And Systemic Symptoms
TNF-α: Tumor Necrosis Factor-α
IL-1β: Interleukin-1β
IL-6: Interleukin-6
CRP: C-reactive protein
HAMD: Hamilton Depression Scale
HAMA: Hamilton Anxiety Scale.
